# Introductory Lecture

**Published:** 1868-10

**Authors:** 


INTRODUCTORY LECTURE.—The Lecture introductory to the term in the Buffalo Medical College will be given by Prof. Sandford Eastman, Wednesday, November 4th, 3 o'clock P. M., in the College Amphitheatre. All interested are respectfully invited to attend.

